# Structured assessment of modifiable lifestyle habits among patients with mental illnesses in primary care

**DOI:** 10.1038/s41598-022-16439-1

**Published:** 2022-07-19

**Authors:** Miriam Pikkemaat, Veronica Milos Nymberg, Peter Nymberg

**Affiliations:** 1 Department of Clinical Sciences, Center for Primary Health Care Research, Lund University, Malmö, Sweden; 2 School of Health and Welfare, Halmstad University, Halmstad, Sweden

**Keywords:** Risk factors, Anxiety, Depression, Lifestyle modification

## Abstract

Patients with mental illness have an increased risk of cardiovascular morbidity. The Swedish-developed Health Dialogue is a pedagogical tool to individualize lifestyle counselling, used in specific age-groups to improve lifestyle habits and decrease mortality, but not tested specifically for patients with mental illness. Patients > 18 years old seeking primary care due to symptoms related to mental illness and diagnosed with depression, sleeping disorders, stress and anxiety, were included. A nurse-led health dialogue was conducted, focusing on lifestyle habits, anthropometric measurements, and blood samples, resulting in tailored advice regarding the individual’s risk profile. All 64 participants had lifestyle areas with increased risk level. Approximately 20% had elevated fasting glucose, blood pressure or cholesterol levels, and over 40% had highest risk level in Waist–Hip-Ratio. 30% were overweight, or physical inactive. The results suggest the need of a larger cohort study with long-term follow up, to establish potentially positive effects on wellbeing, and decreased cardiovascular risk in patients with mental illness.

**Clinical trial registration**: The study was registered at ClinicalTrials.gov January 6th, 2022, registration number NCT05181254.

## Introduction

Both patients with severe mental illness such as bipolarity or schizophrenia and patients with depression and anxiety, have an increased risk of cardiovascular morbidity and mortality compared to the rest of the population, partly related to a higher risk of obesity and diabetes^1–3^. Patients with mental illness in Sweden are managed on different levels, depending on the condition’s severity. Depression, anxiety, stress, or sleeping disorders are common conditions handled by the primary care, while patients with more severe psychiatric diagnosis are treated in psychiatric clinics. It is of great importance to identify high-risk patients early in order to be able to prevent and treat high blood pressure, diabetes, hypercholesterolemia and obesity and to reduce the risk of metabolic and cardiovascular complications^4^.

**Figure 1:**
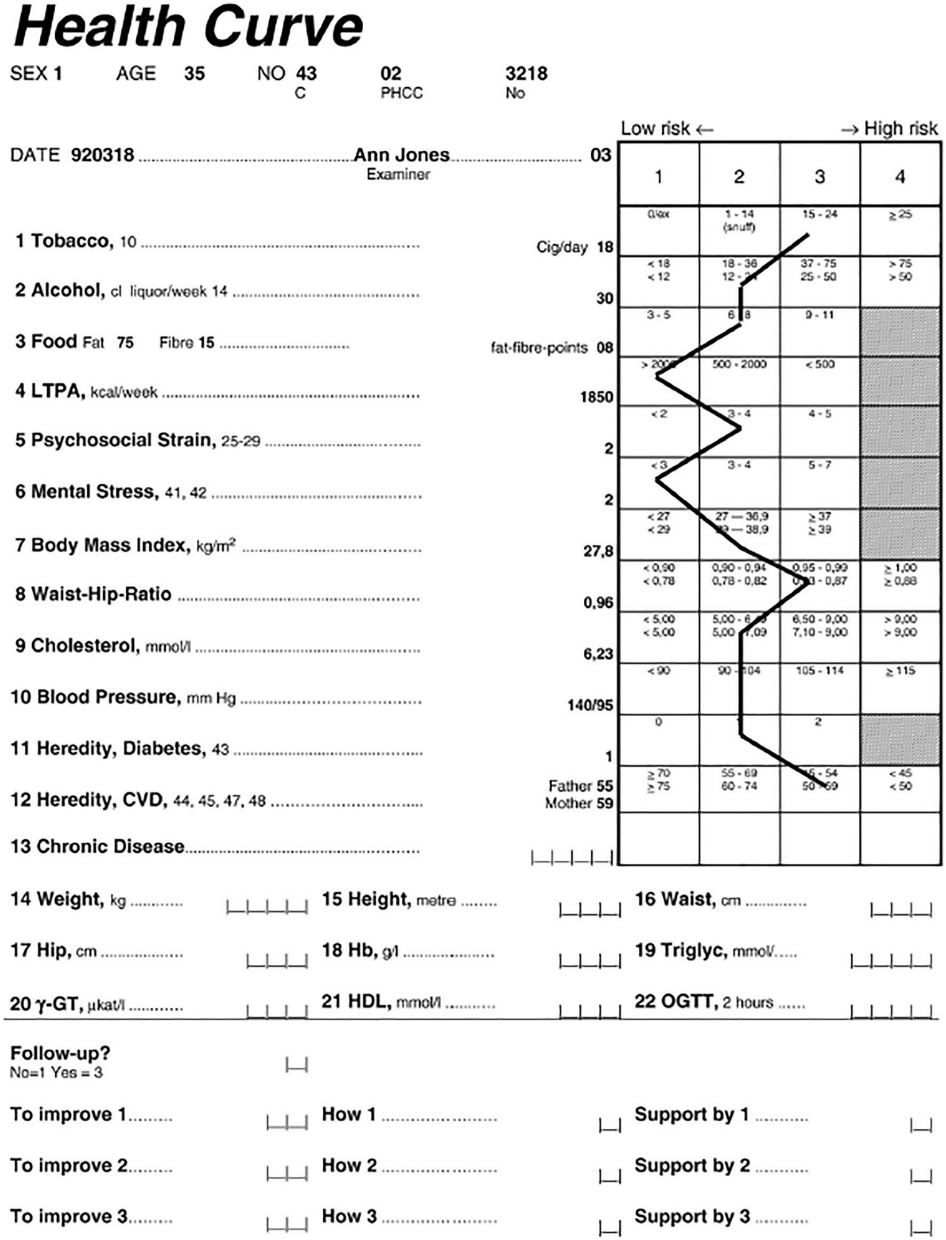
Example of a “Health Curve”, previously published by Ref.^10^.

General practitioners experience that patients with mental illness care less about lifestyle or preventive measures^5^. Meanwhile, the patients want to be encouraged by the health care to change their habits into a healthier lifestyle^6^. Positive life changes and a functioning social network can contribute to a faster recovery from mental illness^7^. There are good examples of lifestyle interventions with physical activity and diet in primary health care for this group of patients, with significant improvements on stress, anxiety and depression^8,9^. There are also indications that individualized programs provide better effects on health and cardiovascular risk factors compared with general lifestyle screening in the population^10^. Targeted health conversations can reduce the risk of developing type 2 diabetes mellitus and cardiovascular disease^11^. To individualize lifestyle counselling in primary care, the Swedish-developed Health Dialogue (Fig. 1, Appendix A) can be used as a pedagogical tool for visualization of the risk score each habit has on the risk of lifestyle related diseases.

The health assessment results in a visual colorful scale (Health Curve) showing a risk assessment from green to yellow, orange and red (Fig. 1, Appendix A) to be used during a health counselling. It is based on both blood sampling such as blood lipids, anthropometric measurements and a web-based questionnaire with detailed questions about dietary habits, physical activity, heredity, smoking, alcohol, stress and mental illness^10,12^. The questionnaire should be filled out before the scheduled health conversation as well as the fasting blood sampling.

In a population-based cohort of 35-year-olds, with an intervention consisting of a health survey based on the Health Dialogue, the participants reported improvements in several lifestyle habits such as less smoking, lower fat intake and higher physical activity^13^. A following study showed that the Health Dialogue is a prognostic tool for assessing the risk of developing diabetes, cardiovascular disease, and cancer^14^. A more recent study with a long-term follow-up showed even reduced mortality after a Health Dialogue^15^.

However, the Health Dialogue is not aimed specifically at patients with mental illness, despite a potentially higher expected benefit of the intervention of this group with an increased risk for metabolic and cardiovascular complications. There are no previous studies on the effect of a systematic approach with the Health Dialogue in patients with mental illness in primary care.

The main aim of this first part of the HEAD-MIP project is to make a base-line description of the patient population included in the project at a primary health care center.

## Methods

### Patients

Patients > 18 years old seeking one primary health care center due to symptoms of mental illness and diagnosed with depression, anxiety, sleep disorders or stress by a general practitioner were offered to participate in the study at the same time with initiation of treatment as usual (psychotherapy or/and medication). At baseline the patients filled out a questionnaire regarding lifestyle habits before the Health Dialogue at the health care center. They were also appointed for fasting blood sampling, measurement of blood pressure, and BMI. A nurse with special training in the Health Dialogue met the patient and provided individually tailored advice based on the patient's unique conditions and the risk profile on the Health Dialogue, such as help with smoking cessation, Swedish version of physical activity on prescription (S-PaP), contact with a dietitian or a physiotherapist. A continued contact with a psychologist or physician was planned if necessary. Patient recruitment took place opportunistically (inclusion after a visit to a doctor or psychologist due to mental illness) and chronologically from the start of the project in February 2020.

### Primary outcome measure

Assessment of the risk profile on the Health Dialogue, self-reported answers about lifestyle (smoking, alcohol consumption, physical activity) and metabolic markers (blood sugar, lipids, BMI, blood pressure, waist-hip ratio) from the Health Curve.

### Data analysis and statistics

Baseline data were analyzed with descriptive statistics. Group comparisons were analyzed with Student’s t-test and χ^2^ test. A p-value < 0.05 was considered significant. SPSS version 27 (IBM Corporation^®^) was used for all statistical analyses.

### Ethics approval

This study was performed in line with the principles of the Declaration of Helsinki. Approval was granted by the Swedish Ethical Review Authority, reference number 2019-04990.

### Consent to participate

Informed consent was obtained from all individual participants included in the study.

## Results

A total of 64 patients were included in the study. All patients had at least one psychiatric diagnosis, 27 patients (42%) had two diagnoses and 8 (12.5%) even a third psychiatric diagnosis. At baseline there were 24 (37.5%) patients diagnosed with depression, 19 (29.7%) diagnosed with anxiety disorder, 22 (34.4%) diagnosed with stress-related disorder (burnout) and 23 (35.9%) diagnosed with sleeping disorder. Seven patients (11%) were already at baseline diagnosed with hypertension, 3 (4.7%) with diabetes, 1 (1.6%) with cardiovascular disease and 3 (4.7%) with hyperlipidemia.

Twenty-one percent of the patients had abnormal fasting glucose values or elevated blood pressure and almost thirty percent had elevated cholesterol levels. Twenty-three percent were very physical inactive and even three quarters of the patients were overweight or obese. Further characteristics are showed in Table 1.

**Table 1:** Characteristics of the study cohort, n = 64.

	Men	Women	Total
Number of individuals, N (%)	17 (26.6)	47 (73.4)	64 (100)
Age at study entry (years), mean (SD)	49.7 (± 12.6)	52.7 (± 7.2)	51.9 (± 14)
WHR, mean (SD)	0.97 (± 0.08)	0.92 (± 0.18)	0.93 (± 0.16)
BMI (kg/m^2^), mean (SD)	29.8 (± 3.9)	27.7 (± 5.6)	28.3 (± 5.3)
Overweight/obesity^a^, N (%)	15 (88.2)	32 (71.1)	47 (75.8)
Fasting s-glucose (mmol/L), mean (SD)	6.5 (± 1.4)	5.5 (± 0.7)	5.8 (± 1.0)
Elevated fasting s-glucose or diabetes glucose-value^b^, N (%)	6 (46.2)	8 (20.5)	14 (21.5)
Systolic blood pressure (mmHg), mean (SD)	130.7 (± 14.1)	124.7 (± 12.8)	126.3 (± 13.3)
Diastolic blood pressure (mmHg), mean (SD)	82.6 (± 7.0)	78.5 (± 8.5)	79.6 (± 8.3)
Elevated blood pressure^c^, N (%)	5 (29.4)	10 (21.3)	15 (23.1)
Cholesterol (mmol/L), mean (SD)	4.4 (± 1.2)	5.0 (± 1.0)	4.8 (± 1.1)
LDL (mmol/L), mean (SD)	2.9 (± 1.2)	3.3 (± 0.97)	3.2 (± 1.0)
HDL (mmol/L), mean (SD)	1.2 (± 0.5)	1.6 (± 0.4)	1.5 (± 0.4)
Triglycerides (mmol/L), mean (SD)	1.4 (± 0.5)	1.3 (± 0.6)	1.3 (± 0.6)
High Cholesterol^d^, N (%)	4 (23.5)	14 (29.8)	18 (28.1)
Hemoglobin, g/L, mean (SD)	154 (± 11.1)	133.2 (± 11.6)	138.0 (± 14.4)
Alcohol, standard drinks per week, mean (SD)	5.2 (± 7.8)	3.2 (± 4.1)	3.73 (± 5.3)
Tobacco use, N (%)	8 (12.5)	5 (7.5)	13 (20)
Very physical active^e^, N (%)	5 (29.4)	9 (19.1)	14 (21.5)
Very physical inactive^f^, N (%)	4 (23.5)	11 (23.4)	15 (23.4)

^a^BMI ≥ 25 kg/m^2^; ^b^> 6.0 mmol/l; ^c^> 140/90 mmHg; ^d^> 5 mmol/L; ^e^risk level 1; ^f^risk level 4.

Risk levels were measured based on the reports on the Health Dialogue formulary, and the results are showed in Table 2. Almost 43% of the participants had the highest risk level (4/red) for WHR and high-risk levels (3/orange or 4/red) on the physical inactivity lifestyle area.

**Table 2:** Risk levels, N (%), measured with the Health Dialogue at baseline.

Risk level	BMI (1–3)	WHR (1–4)	Blood pressure (1–4)	Alcohol (1–4)	Physical activity (1–4)	Tobacco (1–4)	Food (1–3)	Cholesterol (1–4)
1 (green)	33 (52.4)	16 (26.2)	56 (59.8)	50 (78.1)	14 (21.9)	51 (79.7)	37 (57.8)	32 (54.2)
2 (yellow)	26 (41.3)	11 (18.0)	3 (4.9)	7 (10.9)	22 (34.4)	6 (9.4)	18 (28.1)	25 (42.4)
3 (orange)	4 (6.3)	8 (13.1)	2 (3.1)	2 (3.1)	13 (20.3)	7 (10.9)	9 (14.1)	2 (3.4)
4 (red)	N/A	26 (42.6)	0	5 (7.8)	15 (23.4)	0	N/A	0

**Figure 2:**
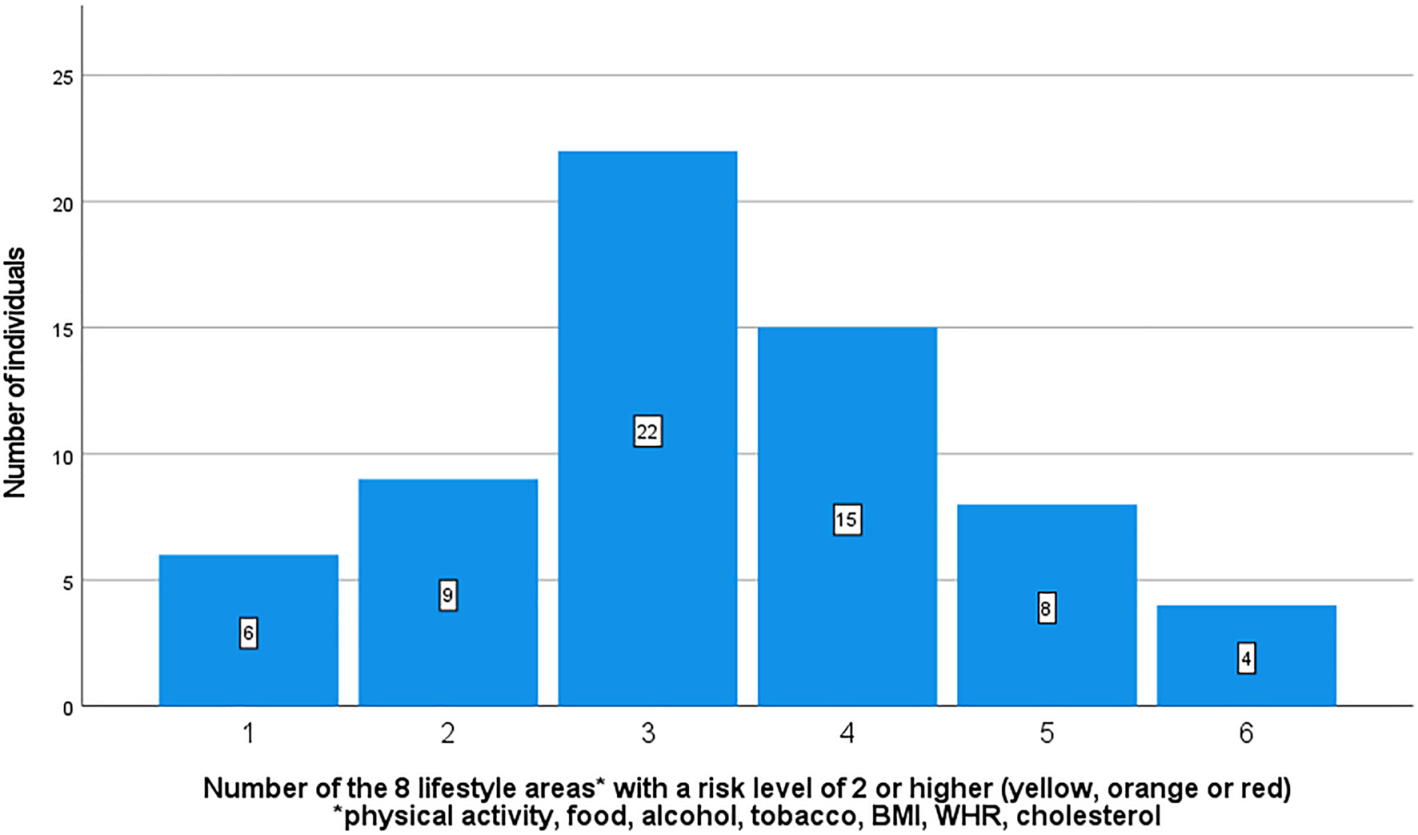
Number of areas with a risk level of 2 (yellow) or higher, (only areas to be treated with lifestyle improvement).

No participant had the lowest level (1/green) in all measured domains. Forty-nine of the 64 individuals (76.6%) had an elevated risk of at least 2 (yellow, orange or red) for three or more lifestyle areas (Fig. 2).

More than half of the participants (54%) had the highest risk level (3/orange or 4/red) on at least one of the studied lifestyle areas (Table 3).

**Table 3:** Number of areas with the highest risk level (3 or 4) (only areas to be treated with lifestyle improvement).

Number of lifestyle domains^a^ with highest risk levels	Individuals (n, %)
1	17 (26.2)
2	14 (21.5)
3	4 (6.2)

^a^Physical activity, food, alcohol, tobacco, BMI, WHR, cholesterol, blood pressure.

**Figure 3:**
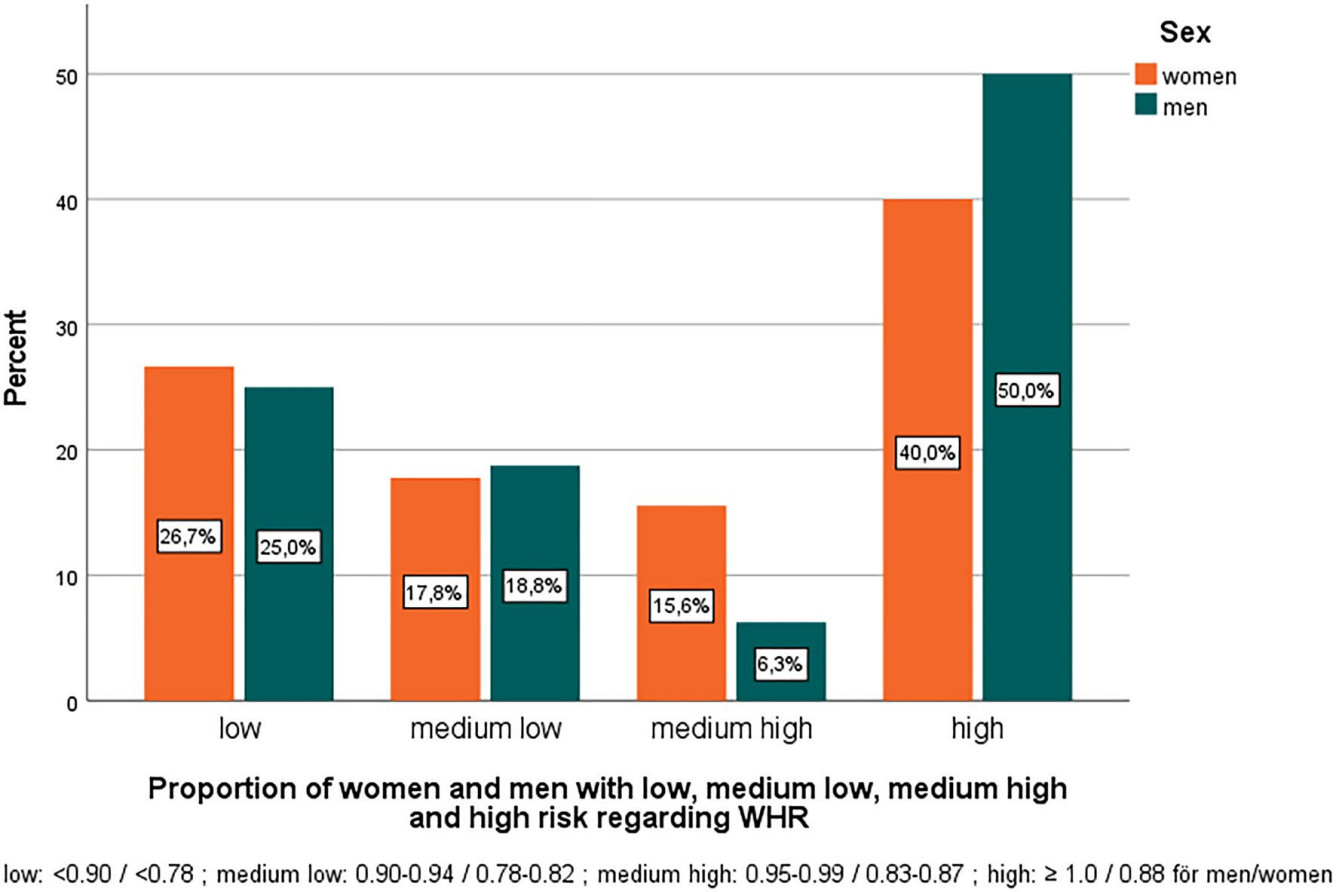
Proportion of women and men with low, medium low, medium high and high risk regarding WHR.

Almost a quarter of both women (23.4%) and men (23.5%) had the highest level (4) for physical inactivity with less than 500 kcal/week. Obesity (BMI ≥ 30 kg/m^2^) was found in 31% of the women and 41% of the men. There was a slightly higher proportion of overweight (BMI 25–29.9 kg/m^2^) among men with 47% compared to women 40%. The distribution of WHR is showed in Fig. 3, showing that half of the men and 40% of the women had the highest level for WHR.

Group comparison analyses (χ^2^ test and Student’s t-test) of higher-risk individuals (defined as having a level of 2 or more in at least 2 lifestyle areas) with lower-risk individuals showed no differences regarding sex (p = 0.30) or age (p = 0.72).

## Discussion

### Main results

In this first part of the HEAD-MIP project, we made a base-line description of the participants, adult patients seeking primary care for mental illness. All participants in our study reported at least one modifiable lifestyle area with increased risk for cardiovascular disease or diabetes. WHR and physical inactivity showed high risk levels in 43% of the studied patients. Other well-known risk factors as increased blood pressure, serum-cholesterol and elevated fasting blood glucose were also found in several participants, even in those without previously known chronic disease.

### Comparison with other studies

Our results are comparable with findings in a screening project using the same method, performed at the same time among 40-year-old individuals in the general population in Southern Sweden, with 411 included participants^16^. In our study 88% of the men and 71% of the women were overweight or obese compared to 71% and 56% respectively in the screened population of 40-year-old individuals. Compared with the general population of 40-year-old, the HEAD-MIP participants showed even a higher proportion of elevated blood pressure (21% women, 29% men versus 2% women, 13% men), abnormal fasting glucose values (21% women, 46% men versus 9% women, 21% men) or physical inactivity (23% women, 24% men versus 13% women, 10% men). The cohorts might not be entirely comparable, since the population in the present study was older (mean age 51.9 years) and all participants were included in the study due to psychiatric diagnoses. However, self-reported data in the screening project of the 40-year-old individuals, showed that 41% of the women and 34% of the men had felt depressed, and roughly half of the participants had sleeping problems during the last year. Many of our study participants in the HEAD-MIP-study, were physically inactive. We believe that a targeted intervention with focus on lifestyle improvement in the group patients with psychiatric diseases might be even more cost effective, as positive life changes as improved diet and increased physical activity have been showed to increase remission rates of non-psychotic mental illness^17,18^.

In older adults it has been revealed that lifestyle habits such as smoking or low levels of physical activity can indicate mental illness such as depression^19^. The accumulation of several bad habits, smoking, high alcohol consumption, inactivity and poor nutrition, increases the risk of depression. It has also been shown that psychological wellbeing is influenced by lifestyle habits, with reported higher grade of psychological wellbeing among those who had a healthy diet, were physical active and nonsmokers^20^.

Despite the small sample size of this study, it confirms the hypothesis that this group of patients needs closer attention regarding lifestyle areas that might affect the psychiatric wellbeing as well as increase the risk to develop diabetes or cardiovascular disease. Detecting patients with the highest risks allows targeted interventions aiming to improve mental wellbeing but also lowering the cardiovascular risk and complications. We could show that more than 20% had either elevated blood glucose, cholesterol, blood pressure or several altered measurements. Even if some of the participants had previously diagnosed diabetes, hyperlipidemia or hypertonia, the Health Dialogue helped to discover impaired lab results even in other participants without previously known chronic disease.

The results indicate that Health Dialogues might be an effective method of detecting elevated blood glucose and blood pressure in patients with mental illness. Early detection enables follow-up combined with treatment using lifestyle interventions and if necessary, even with medication, to avoid or at least postpone both a chronic disease diagnosis and later complications.

To our knowledge, the use of a Health Dialogue has not previously been studied in a cohort of patients with mental illness. In the second part of the HEAD-MIP study we are aiming to evaluate the effect of the Health Dialogue on lifestyle habits among patients with mental illness.

### Strengths and limitations

The study cohort is limited to a small amount sample size of patients recruited at one primary health care center, therefore with low generalizability. A larger cohort with higher sociodemographic representation and several primary health care centers is planned. The screening project with 40-year-old individuals using the same method in the same primary care context showed good feasibility in the general population as well as cost effectiveness. A major limitation in our study is that we were not able to study the inclusion rate, due to the recruiting method. Several caregivers (physicians, nurses, and one psychologist) included patients opportunistically after the initial contact at the primary health care center due to psychiatric illness. However, we believe that the inclusion method might have included a higher rate than screening with invitation letter in this group of patients, a strength of the study is therefore the opportunistic patient inclusion, with patients being recommended a lifestyle assessment by the caregiver, minimizing the risk for inclusion bias. Severe mental illness was not an exclusion criterion. However, patients with severe mental illness are not usually managed in primary care in Sweden and none were therefore included in this study. Our results are therefore not transferable to this patient group, and this is a limitation of the study. Another limitation of the study is lack of data on socioeconomical status of the participants. Socioeconomic status is associated with increased prevalence of tobacco, physical inactivity, and poor nutrition^21^. Greater financial stress may also be associated with increased rates of mental illness. Even if patients were listed on the same health care unit, meaning that the socioeconomic status is expected to be approximately the same on group level, individual socioeconomic differences might have existed.

Several participants in our study had increased levels of blood pressure and fasting blood-glucose. Even if these surrogate measurements might indicate possible development of chronic disease as high blood pressure or diabetes, diagnosis needs several repeated valid values. Another limitation is lack of data about medications with potential side-effects as weight gain such antipsychotics or some anti-depressants. Patients with pre-existing conditions as diabetes or hypertension diagnosed prior to the inclusion in the present study have probably received qualified lifestyle counselling by health care personnel at the point of diagnosis. Due to the limited number of patients in the present study, no subgroup analysis was made. Future studies should take this aspect in consideration.

## Conclusion

The Health Dialogues in primary care patients with mental illness showed that all participants had at least one lifestyle area with increased risk level to develop diabetes or cardiovascular disease. In addition, most of the participants were obese, physically inactive, or both. We suggest that this patient group should receive closer attention and argue that a larger cohort with long-term follow up is highly motivated.

## Supplementary Information

Supplementary Information.

## Data Availability

The datasets used and/or analysed during the current study available from the corresponding author on reasonable request.
