# Supplementary figures and images for: Structured assessment of modifiable lifestyle habits among patients with mental illnesses in primary care
Source: Sci Rep. 2022 Jul 19;12:12292. doi: 10.1038/s41598-022-16439-1 (PMC9296453; doi:10.1038/s41598-022-16439-1)

Appendix A


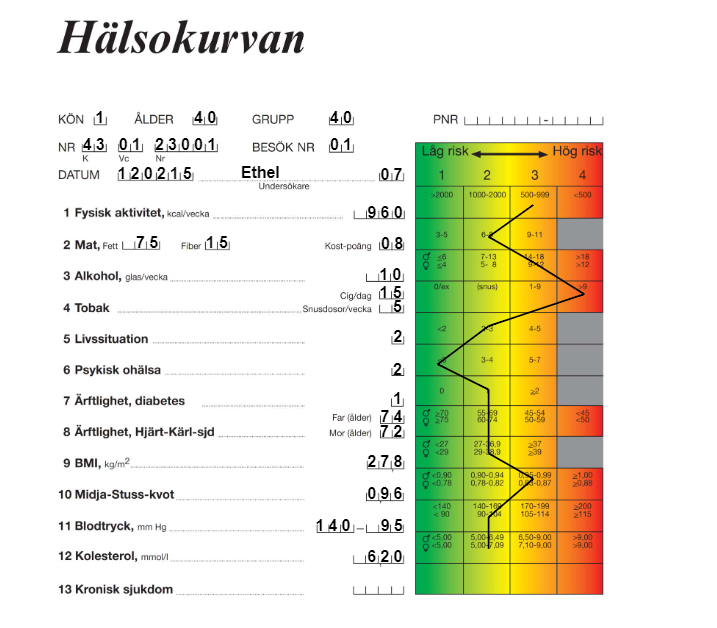


Figure A. Health Dialogue, the summary page (coloured, original Swedish version)

Supplement: Supplementary file 1 — Supplementary Information. [file 41598_2022_16439_MOESM1_ESM.docx]
